# The drug-minded protein interaction database (DrumPID) for efficient target analysis and drug development

**DOI:** 10.1093/database/baw041

**Published:** 2016-04-06

**Authors:** Meik Kunz, Chunguang Liang, Santosh Nilla, Alexander Cecil, Thomas Dandekar

**Affiliations:** ^1^Functional Genomics and Systems Biology Group, Department of Bioinformatics, University Wuerzburg, Biocenter, 97074 Wuerzburg, Germany; ^2^Bioinformatics & Biostatistics, Helmholtz Center Munich, Institute of Experimental Genetics, Genome Analysis Center, Ingolstaedter Landstrasse 1, 85764 Neuherberg; ^3^Present address: Wagnerstrasse 15 97080 Wuerzburg, Germany

## Abstract

The drug-minded protein interaction database (DrumPID) has been designed to provide fast, tailored information on drugs and their protein networks including indications, protein targets and side-targets. Starting queries include compound, target and protein interactions and organism-specific protein families. Furthermore, drug name, chemical structures and their SMILES notation, affected proteins (potential drug targets), organisms as well as diseases can be queried including various combinations and refinement of searches. Drugs and protein interactions are analyzed in detail with reference to protein structures and catalytic domains, related compound structures as well as potential targets in other organisms. DrumPID considers drug functionality, compound similarity, target structure, interactome analysis and organismic range for a compound, useful for drug development, predicting drug side-effects and structure–activity relationships.

**Database URL:** http://drumpid.bioapps.biozentrum.uni-wuerzburg.de

## Introduction

New analysis technologies have contributed to huge volumes of molecular data. Numerous databases have been developed to explore these (1–5) with complementary focus on protein interactions, side effects, or drug information.

The drug-minded protein interaction database (DrumPID) has been designed for researchers to quickly obtain custom, tailored information on drugs and protein interactions with the idea to rapidly understand and screen related compounds for their effects in protein interaction networks considering related organisms. It fills here a niche between the current databases, quite useful to explore potential antibiotic lead structures, optimizing predictions from animal tests and better explore the chemical space around a compound together with the protein interaction networks affected. For each capability DrumPID makes direct calculations based on the chemical properties of the drug, collating and comparing information from several databases, as well as its own stored data.

A broad user interface is displayed on multiple windows, allowing the user to compare drug-centered and protein-centered queries at the same time. Multiple windows also allow the user to study and compare targets and interactions between different drugs. Moreover, the acquired information can be further analyzed with biological software systems, such as cytoscape and embedded plugins. Besides the drug name, chemical structures (SMILES notation) and affected proteins (as potential drug targets) can also be queried. Furthermore, a combination of querying options allows the user to derive information as well as screening for drugs and drug families, their chemical properties, involved protein networks, organism-specific protein interactions and general protein families. SMILES strings help in posing queries. They are easily placed in large windows. There is an intuitive auto-completion function as well as automatic removal of blanks. Additional search options cover information on indications and pathway maps. Moreover, an implemented similarity search also enables the identification of similar drug molecules for SMILES notations and allows further analyses, e.g. potential targets, especially for new synthesized compounds.

## Materials and methods

FDA-approved drugs from the DrugBank database (1, 2) were used as the backbone for generating chemical compound information. The data extraction began by downloading sdf- and SMILES-files of all FDA-approved drugs (the current DrumPID version includes 1383 FDA-approved drugs, in addition, >5000 FDA and non-approved drugs are made available in the accepted manuscript). These files contain—among other information—the atomic 3D structure for each compound. Based on these data, we calculated specific chemical properties (molecular and atomic descriptors) using the cheminformatics R package rcdk (7). Additional pharmacological and drug indication information were taken from DrugBank (1, 2) and Drugs.com (http://www.drugs.com/) by warehousing existing information and drug links.

### Drug target and pathway information

For each drug, we downloaded protein targets and corresponding pathways from the DrugBank (1, 2) and KEGG (14) databases. In addition, based on the sequence for each drug target, we performed an orthologous group search (COG/KOG; 8) using our in-house COGMaster from the JANE package (6).

### Analyzing structural information

Based on the SMILES notation we calculated the corresponding drug structure (as SVG output file) using the command-line utility indigo-depict from the cheminformatics indigo toolkit (http://lifescience.opensource.epam.com/indigo/). Moreover, we implemented an additional perl script which converts SMILES strings into a PDB structure file for download (opening in a new popup window).

### Data storage and implementation

**Figure 1.**
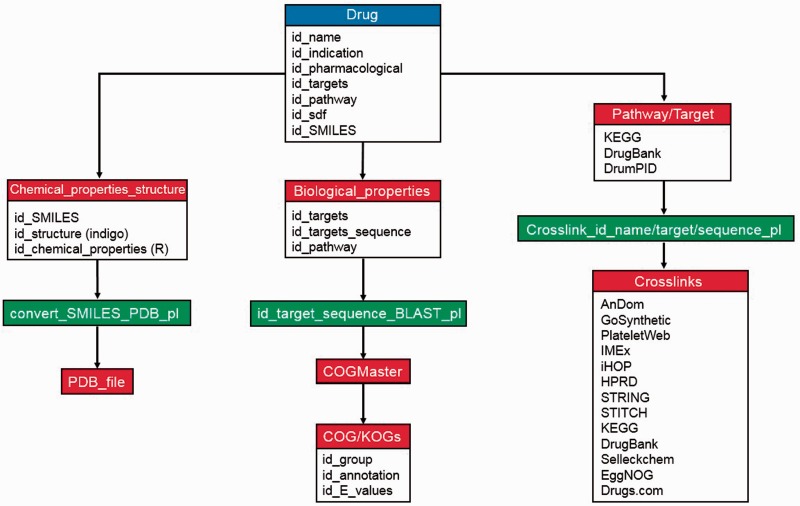
Maintenance and database scheme of DrumPID database. The workflow illustrates detailed maintenance and update procedures of DrumPID database. We update drugs and their properties each month, the steps are shown above. Once a new drug is added into DrumPID database (blue box), own calculation procedures (red boxes) are carried out manually, next all structure file conversions, related crosslinks and bridging information are automatically generated by scripts (green boxes), i.e. COGMaster from JANE package (6) and other Perl scripts.

Regarding data storage and implementation, all downloaded information (KEGG, DrugBank and Drugs.com databases) and calculated data (e.g. molecular weight, Lipinski’s rules and COG/KOGs) were internally stored and warehoused into a MySQL database (in which each drug is given a unique identifier) to benefit from the advantages of a relational database. These include efficient data management, easy data-updates and rapid accessibility for our search engine. There is an inbuilt logic of database preference and information provision (e.g. drug–protein interaction and crosslinks; palette view), which is implemented mainly in PHP (see Figure 1 for database scheme).

DrumPID offers multiple search categories with multiple queries and keywords (including data mining applications) and query assistance (heuristic auto-completion function, demonstration queries; tutorial in supplementary material). In addition, we implemented a similarity search for SMILES notations using the fingerprint function (‘tanimoto’ similarity metric method, threshold >0.66) from the cheminformatics R package rcdk (7). DrumPID warehouses and compares several drug information and interactome databases (including back-links to the original database). Furthermore, it gives various crosslinks to other databases with information on: affected COG/KOGs [STRING (4) and EggNOG (16) database]; large-scale and organism-specific interactions [HPRD (12), iHop (13), STRING (4), IMEx (15) and our in-house PlateletWeb (11) database]; detailed structure prediction [with our in-house AnDom software (9)]; detailed function prediction [with our in-house GoSynthetic database (10)]; and identification of potential inhibitors/activators of target proteins with the compound screening library Selleckchem (http://www.selleckchem.com/; online drug repository catalogue). For detailed information and workflow, see Figure 1 and Table 1.

**Table 1.** Search categories and output overview^a^

Opportunity	Description
Search category	
Indication	This search category will check against all indications of all drugs in the database. It will be helpful to find out the best possible drug against a given pathological condition
Associated pathogen	This search category is for disease-caused organism and gives all drugs against the pathogens
Drug name	A plain-text search if the name of the studied drug is known
SMILES	This category search for the SMILES of a drug. This search category will be the best way to find similar drugs in the database
Affected protein	All drugs deposited in the database will be checked for their respective effects on target proteins and will be helpful to search for drugs which affect a specific protein
SMILES similarity	This category search for similar substructures of a SMILES in drugs based on Tanimoto similarity score matrices. This search category will be the best way to find similar drugs in the database showing, e.g. same targets (Results are shown in a separate table with threshold >0.66.)
Result table	
Generic name	The name of the drug is given
Drug ID	The corresponding Drug ID is given
External links	Links to external databases (e.g. DrugBank and Selleckchem) is given to get additional information
Pharmacological properties	The pharmacological description of the drug according to DrugBank is given
Indication properties	Information about the drug indication according to DrugBank is given
Structure	The structure of the drug is shown
SMILES and PDB structure	The corresponding SMILES for the drug is given and also a function to convert the SMILES into PDB structure files is implemented
Chemical formula	The drug chemical formula is indicated
Atom count	The atom count of the drug is calculated
Mass	The molecular weight (part of the Lipinski’s rule of five) of the drug is calculated
H-bond donor count	The H-bond donor count (part of the Lipinski’s rule of five) of the drug is calculated
H-bond acceptor count	The drug H-bond acceptor count (part of the Lipinski’s rule of five) is calculated
logP	The logP (part of the Lipinski’s rule of five) of the drug is calculated
Ring count	The drug ring count is calculated
Polar surface area	We calculated the drug polar surface area
van-der-Waals surface area	The van-der-Waals surface area of the drug is calculated
Target pathways	The targeted pathway of the drug is given including a crosslink to the corresponding databases DrugBank and KEGG (by moving the mouse above).
Protein binding	The percentage of the protein bound is given
Protein interactions	The target (from DrugBank and KEGG) of the drug is given including crosslinks to PlateletWeb- (protein interactions in platelets but also in general in human cells), AnDom- (3D structure prediction and interactions) and GoSynthetic-Database (functional interaction predictions) as well as to the public HPRD-, iHOP-, STRING-, KEGG and IMEx-Database. This allows a detailed examination of interactions in different aspects, putting the drug into its interaction context (see tutorial)
Ortholog group of target protein	Each drug target is investigated with an Orthologous group search (COG/KOG). The resulting COG/KOG is shown with their annotation and *E*-value. The user can follow hyperlinks to the GoSynthetic-, STRING- and EggNOG-Database for further information about the COG/KOG (by moving the mouse above)

a Database logic shows all active links with original database information, use case and tutorial. Demonstration examples at the Web interface of DrumPID illustrate the database usage.

## Results and discussion

**Figure 2.**
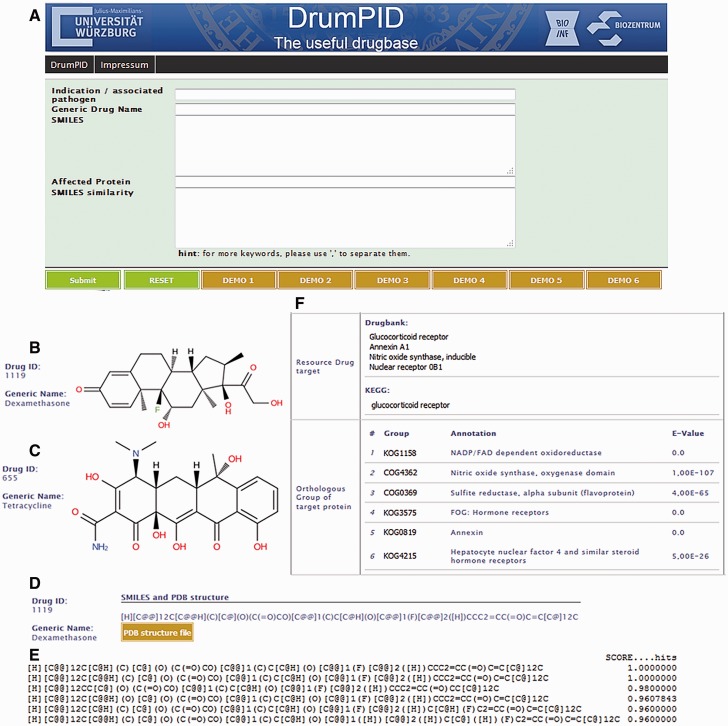
DrumPID search capabilities. DrumPID allows the user to explore potential antibiotic lead structures, optimizing predictions from animal tests or explore the chemical space around a compound together with the affected protein interaction networks. For each capability, DrumPID makes direct calculations based on the chemical properties of the drug as well as collating and comparing information from several source of databases (database logic rules show all original database sources available) and its own stored data (see text for details). (**A**) Web interface. DrumPID allows to search for Indications and associated Pathogens, generic drug names, SMILES, drug-affected proteins as well as similar substructure of SMILES. (**B**) Drug indication query (hematological disorder). Example: the drug Dexamethasone with corresponding structure and Drug ID, scroll down for more information (not shown). (**C**) Pathogen query. Example: drug Tetracycline (structure) against *Borrelia burgdorferi (B. burgdorferi)*. There is further information on treatment, drug usage as well as chemical and biological properties (not shown). (**D**) SMILES search. Example: [H][C@@]12C[ C@@H](C)[C@](O)(C (=O)CO)[C@@]1( C)C[C@H](O)[C@ @]1(F)[C@@]2([H])CCC2  = CC(= O)C  =  C [C@]12C. The resulting drug Dexamethasone is shown. Furthermore, SMILES notation is converted into PDB structure files, which enables further studies of the compounds, e.g. docking studies. (**E**) SMILES similarity search. In addition, to identify drugs consisting similar substructures, a similarity search for SMILES is possible (Tanimoto similarity score > 0.66). For example, using the SMILES [H][C@@] 12C[C@@H] (C)[C@](O)(C( =O)CO) [C@@]1(C)C[C@H] (O)[C@@]1(F)[C@@]2([H])CCC2 = CC(=O)C = C[C@]12C calculates Dexamethasone and Betamethasone with a similarity score of 1 as top hits (here hits >0.96 are shown). (**F**) Protein interactions. For each drug, known targets and pathways are given (including source scheme; here only targets shown). For all targets there is Ortholog group search (COG/KOG) including annotations and *E*-values. Furthermore, output entries carry links including other interaction databases (PlateletWeb, AnDom, GoSynthetic, HPRD, iHop, STRING and KEGG) are available (not shown). Example: Glucocorticoid receptor gave 37 results, four Protein interactions and six Ortholog Groups for the drug Dexamethasone. (For more details, see text and tutorials in supplementary material.)

Queries place the drug into its protein interaction context considering indication/associated pathogen, generic drug names, compounds (SMILES notations) as well as affected proteins and networks (Figure 2 and Table 1). Each drug in the database is given a unique identifier whereby drugs can be readily queried and investigated with our platform. The search strategy allows users to search for them by any relevant information from these entry points, for instance: generic drug name, target protein, chemical structure (SMILES annotation), as well as indication or pathogen organism. Notably, we provide a similarity search option for SMILES notations. Simply by putting a SMILES string into the search field, our implemented function screens for similar drug SMILES in our database (results are represented in a table including the substring matching and calculated similarity scores). In addition, our search engine offers multiple queries such as term-based queries with wild cards, auto-completion, symbol-based queries for structures joining multiple searches and categories as well as refined queries with multiple keywords in one or more categories separated by comma (‘,’). The tutorial (see supplementary material) and demonstration queries guide the user.

DrumPID contains 1383 FDA-approved drugs, 4951 proteins, 4078 ortholog groups (clustered according to 993 unique COG/KOG, 21120 orthologs from 67 different organisms) and over 1 million different protein interactions (various organisms); in addition, stored data from >5000 FDA and non-FDA approved drugs are made available (upon acceptance of the manuscript). Up to 50 results are shown in one page, indexed entry numbers allow to browse all results. The result page is divided into different sections:

- identifiers (DrumPID ID, generic name and structure (SVG figure; SMILES and downloadable PDB structure; external drug links to DrugBank, KEGG, STITCH, Drugs.com and the Selleckchem databases appear according to the information available),
- biological properties (targeted protein interactions and pathways including source scheme),
- protein binding affinity and orthologous groups (including E-value) of targeted proteins,
- chemical properties (e.g. Lipinski’s rules), pharmacological information and indications (see Figure 2 and Table 1).

In addition, pathway cards from KEGG are directly shown (zoom out by mouseover). Moreover, for protein interactions we provide crosslinks to organism-specific interactome databases [HPRD (12), iHop (13), STRING (4), IMEx (15), EggNOG (16) and in-house PlateletWeb (11) database], structure prediction database AnDom (9) and molecular process analysis database GoSynthetic (10) (see Table 1). Thus, users can directly obtain the whole interaction context and/or study specific interactions in more detail simply by following the crosslinks.

Compared with other databases, the DrumPID interface is easy to navigate and handle, and results are all quickly accessible from within one page. Alternative databases excel in complementary aspects: ChEMBL (3) regarding compounds (over 1.7 million); Cambridge structural database (http://www.ccdc.cam.ac.uk/pages/Home.aspx; over 800 000) and ChemSpider (17; over 35 million) regarding structures, whereas DrugBank database (1, 2) links drug data and target information. DrumPID starts with structural information (e.g. SMILES notation, PDB structure, chemical properties) but it excels in target structure and interaction predictions to put drugs and target proteins into a detailed interaction context. For each task, data from at least two sources are combined and the combination of several chemical algorithms, interaction predictions and clustering guarantees superiority to just one method or only one of the used data repositories. Interaction data from the drug protein interaction bases DrugBank (1, 2) and KEGG (14) are combined; regarding drugs, data from DrugBank (1, 2) and KEGG (14) as well as Drugs.com (http://www.drugs.com/) are combined; regarding structure, function and interaction, predictions from AnDom (9) and GoSynthetic (10) are combined plus direct calculations of COGMaster (6) and disease information from DrugBank and data crosslinks, e.g. to STRING and IMEx (1, 2, 4, 15). Thus various protein interaction databases allow to screen and add potential interactions, e.g. with a disease in mind as well as development of organism-specific target protein structures (e.g. for antibiotics) by suitable query options. External links (e.g. back-link to original database and Selleckchem catalogue; http://www.selleckchem.com/) offer users the option to get further detailed information on a drug depending on the scientific interest.

Moreover, for each drug target the corresponding orthologous group (COG/KOG) is calculated. The COG/KOGs are calculated comparing over all complete genomes all proteins belonging to the same gene family and labeling them with the same number to indicate that they belong to the same cluster of COG/KOG sequences. The COG/KOGs help to identify all proteins of this family occurring in the same organism. For a given drug, this indicates how easy the drug will reach other proteins also belonging to the same family as the target protein, which gives a good first estimate for potential side-effects (e.g. a large receptor family, where each receptor can also respond to the same drug). Furthermore, comparing the same COG/KOG over a range of organisms allows the prediction of drug effects for a whole clade or even larger groups. This is, for instance, useful when estimating how broad acting an antibiotic drug will be. Furthermore, the COG/KOG also allows the user to see the complete variation of the target protein family over all organisms. Together with some pharmacological data (of course only if available) this allows a first estimate of quantitative structure–activity relationship for the target protein COG/KOG family (e.g. comparing N- and C-terminal variation in different organisms).

In addition, our substructure similarity search option helps users to find a corresponding substring for a SMILES input and allows further analysis, e.g. drug target analysis. Thus, the search option helps to obtain information of the drug functionality which is of interest especially for previously unknown and new synthesized compounds. Moreover, due to the fact that substrings can influence various targets, the similarity search also allows for an input drug the SMILES-based identification of additional side-targets based on the substrings, which might be helpful for drug development, e.g. off label use or new treatment options by adding new side chains.

### Application use cases

The following application examples illustrate how DrumPID can be used to find drugs for a target and rapidly explore its target interaction context. The examples illustrate *in silico* search modes of different types and are specific predictions generated from DrumPID for each use case. They were specifically generated by us to show the potential of DrumPID based on typical scientific questions and challenges in drug research. Our DrumPID allows an integrated drug-minded view at the same time, this makes investigation of drug–protein interactions more specific, e.g. functional COG/KOGs information in different organism, large-scale and tissue specific interaction screens such as in platelets (PlateletWeb) and other tissues (e.g. GoSynthetic and STRING database) as well as experimental (e.g. IMEx and STRING database) and predicted protein interactions (STRING database) (see also tutorial supplementary material). A natural limitation of DrumPID is the amount and type of data stored, in particular searches will only work according to the key words given and suitable matches to data stored. For a good search, a detailed analysis combining several steps and key words is best (details in supplementary material tutorials). A further option besides querying with key words (indications, proteins, etc.) are searches with SMILES strings according to chemical similarity.

### Use case 1: drug information

**Figure 3.**
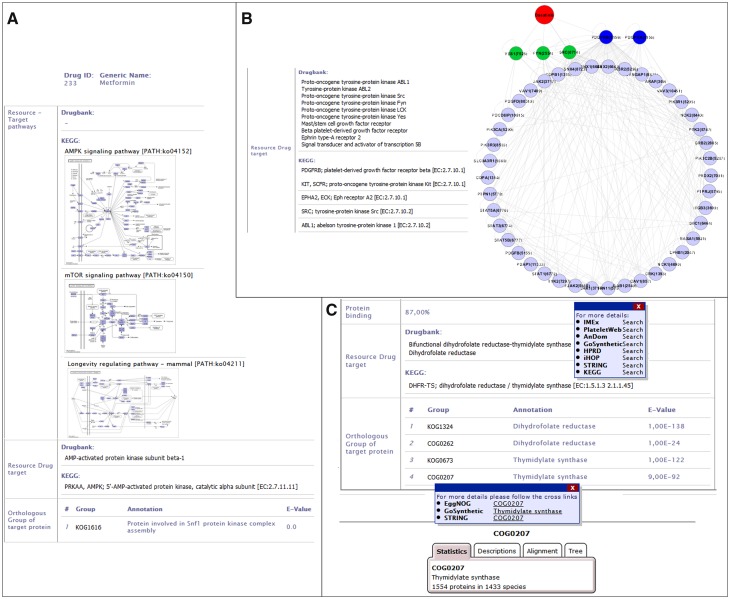
DrumPID use case examples. (**A**) Drug: DrumPID example to find a drug activating a target protein. (**B**) Target protein: DrumPID example to study target proteins in a cell-type-specific context. (**C**) Organism: DrumPID example to identify target proteins across various organisms. (DrumPID screenshots for illustration, detailed explanation of the shown data is in the text.)

The standard use case is to get information on a specific drug or to screen a database to find available drugs for a target protein. For example, we are interested in a drug to activate the AMP kinase (AMPK) in cancer cells. For this, users can screen DrumPID for tyrosine-protein kinases (category affected protein ‘AMP-activated protein kinase’; four results). We will focus on one of these, Metformin (see Figure 3A), which can be further explored for instance regarding the chemical effect (activator, inhibitor) and the network effects. Users can readily identify that Metformin is mainly used to treat diabetes. Furthermore, by considering the whole protein interaction and pathway context users find not only a link to the mTOR (mechanistic Target of Rapamycin) pathway but also an additional link to the AMPK- and longevity regulating pathway (see Figure 3A, AMPK target considered from KEGG and DrugBank source; whole AMPK interaction context can be obtained by hovering over the pathway map and/or by following crosslink, e.g. to KEGG or IMEx, not shown here).

This connection, as well as other such interactions, alludes to Metformin’s recently discovered life prolonging abilities in diabetic-patients with still open potential in healthy individuals (18). Moreover, AMPK is downregulated in cancer and known not only to activate p53 to induce apoptosis, but it also inhibits the mTOR pathway. The latter is associated with metabolism, proliferation and angiogenesis. For this, Metformin could be a potential activator of AMPK signaling (see also link to Selleckchem catalogue; here not shown) to influence apoptosis and the mTOR pathway (see Figure 3A, mTOR pathway ko04150). Thus, Metformin will not only affect diabetes but may be of interest, e.g. as off-label use for cancer treatment in AMPK mutated tumors, which can further be experimental tested by users.

### Use case 2: protein target

This example shows the identification of a drug to target a specific protein, for example the platelet-derived growth factor receptor beta (PDGFRB). For this search, DrumPID (search category ‘affected protein: platelet-derived growth factor receptor’) identifies four drugs. Simply by browsing DrumPID users can see that these drugs not only target PDGFRB but also have several other targets. It is well known that the PDGFR signaling (PDGFRB-PDGFRA dimer) is activated by binding of PDGF ligands and involved, e.g. in wound healing and post-infarction cell repair. However, for users it is important to study exactly the network connectivity of such multi-target drugs in a cell-type-specific context, e.g. in platelets. DrumPID can support such specific analysis: following the target link to our in-house PlateletWeb database (other tissue-specific contexts can also be studied using STRING database and other cell types, not shown here), users can find out that PDGFRB has 46 platelet interacting proteins. Furthermore, there are 20 non-platelet interacting proteins in other human tissues (in total 66 interacting partners; see target link to PlateletWeb). Figure 3B illustrates the DrumPID network of PDGFRB in platelets using only 46 validated platelet interacting proteins of PDGFRB from our in-house database PlateletWeb (downloaded and visualized with Cytoscape software; PDGFRB–PDGFRA dimer shown in blue; Src, Yes1 and Fyn in green; Dasatinib in red; interactions in grey). By considering the drug targets from DrumPID, users can easily find a link between PDGFRB, Src, Yes1 and Fyn indicating Dasatinib (targets all four) as best potential drug influencing not only PDGFRB but also other interaction partners around the network in platelets. Thus, DrumPID allows users to rapidly investigate the interactome of drug targets and side-targets in a cell-type-specific manner, e.g. to find best drug candidate for further analysis.

### Use case 3: organism-specific drug effects

DrumPID is also useful for more specialized scientific questions such as organism-comparative queries, for instance, identification of potential targets and drugs which specific inhibit the parasitic DNA replication but do not affect humans. Figure 3C shows how users can rapidly identify and analyze the target of the thymidylate synthase in trypanosomes. One current treatment option for trypanosomiasis (see sleeping sickness and/or trypanosomes in DrumPID) is Suramin; however, its mechanism is unknown. Treatment options mainly focus on blocking the cell cycle of trypanosomes, e.g. thymidylate synthase. Such drugs can be easily identified by DrumPID: Simple by searching for thymidylate synthase (see Figure 3C), users will find 13 drugs (not shown here; none of them for trypanosomiasis). However, the implemented orthology search identifies the COG0207 (1554 proteins in 1433 species) as an orthologous group of the target thymidylate synthase (see Figure 3C). As a next step, users can rapidly identify the corresponding protein of the thymidylate synthase across various organisms by following the crosslinks, e.g. to STRING database (e.g. trypanosomes *Trypanosoma brucei (T. brucei)* AAZ12612; *Trypanosoma cruzi (T. cruzi)* DHFR-TS and XP_819618.1; here not shown). The identified protein can be further analyzed, e.g. regarding the protein structure and/or the interaction network (not shown here). Thus, users are not only able to explore the specific target but also design new experiments and therapeutic agents for trypanosomiasis, e.g. which are highly selective against *T. brucei*, but show no effect in human cells.

A drug design challenge we study intensively is antifungal treatment in *Aspergillus fumigatus* infection. DrumPID readily identifies here drugs for promising protein targets. We illustrate this for the two metabolic enzymes ergosterol (easier task: no ergosterol metabolism in humans) and riboflavin synthase (challenge task: humans have the enzyme, too). Searching DrumPID for ergosterol identified six drugs including Amphotericin B and Natamycin well known in the treatment of *Aspergillus* infection. This validates the approach, as these drugs are even FDA approved. Furthermore for riboflavin synthetase, the suggestion by DrumPID concerns riboflavin (FDA approved, vitamin B2) and hence fungal-specific antimetabolites (modifying riboflavin) that bind exclusively *A.* *fumigatus* riboflavin synthetase. For this, detailed analyses are necessary: differences to the human version in the *A.* *fumigatus* riboflavin synthetase are made visible from COG/KOGs in DrumPID. Moreover, users get further information, e.g. regarding the pathway and orthologous groups, help in analyzing differences between organisms which then enables to develop a drug specifically targeting only fungal metabolic processes but no human processes (see supplementary material for details).

### Advanced use cases

Advanced use cases include:

- The analysis of protein targets for which currently no drug is available. Using our COGMaster function, users get the functional COG/KOG classification and corresponding drugs for the COG/KOGs from DrumPID, which allow, e.g. functional pathway analysis to find differences/similarities in human and model organisms. Predicted targets are then available for further experimental tests.
- For new synthesized compounds with unknown functions, users can use the similarity search function, which rapidly calculates the corresponding substring for a SMILES input. Thus, users get first potential functional information and can explore potential antibiotic lead structures, optimize predictions from animal tests or explore the chemical space around a compound together with the affected protein interaction networks.

## Conclusion

The DrumPID allows the user to rapidly understand and screen compounds for their effects in protein interaction networks, considering a bundle of interactome databases and algorithms, related organisms and searches for disease indications. It is useful for exploring potential antibiotic lead structures, optimizing predictions from animal tests and exploring the chemical space around a compound together with protein interaction networks. Users may study individual pathways or protein interactions, as well as potential targets in various organisms. Protein structures are rapidly analyzed, including catalytic domains and SCOP classification as well as structure prediction (AnDom). DrumPID will be updated every month.

## Supplementary data

Supplementary data are available at *Database* Online.

Supplementary Data
