# Supplementary material for: The drug-minded protein interaction database (DrumPID) for efficient target analysis and drug development
Source: Database (Oxford). 2016 Apr 6;2016:baw041. doi: 10.1093/database/baw041 (PMC4823820; doi:10.1093/database/baw041)
Supplement: Supplementary Data [file supp_2016_baw041_index.html]

Supplementary Data 

# The drug-minded protein interaction database (DrumPID) for efficient target analysis and drug development

## Supplementary Data

files

- Supplementary Data - doc file
