# Supplementary material for: The drug-minded protein interaction database (DrumPID) for efficient target analysis and drug development
Source: Database (Oxford). 2016 Apr 6;2016:baw041. doi: 10.1093/database/baw041 (PMC4823820; doi:10.1093/database/baw041)
Supplement: Supplementary Data [file supp_baw041_DrumPID_suppl_Rev.doc]

| The Drug-minded Protein Interaction Database (DrumPID) for efficient target analysis and drug development  Meik Kunz^1,§^, Chunguang Liang^1,§^, Santosh Nilla^1^, Alexander Cecil^2,*^**,** Thomas Dandekar^1,*^  ^1^Functional Genomics and Systems Biology Group, Department of Bioinformatics, Biocenter, Würzburg, Germany.  **^2^**Genome Analysis Center / Institute of Experimental Genetics Building 34, Room 336; Helmholtz Zentrum Muenchen; Deutsches Forschungszentrum fuer Gesundheit und Umwelt (GmbH) Ingolstaedter Landstr. 1; 85764 Neuherberg  ^§^Authors contributed equally to this work.  *corresponding authors    **SUPPLEMENTARY MATERIAL:**   - **Tutorial I: Using DrumPID database** - **Tutorial II: Putting the drug into its protein interaction context** - **Tutorial III: Application study anti-fungal drugs** - **Declaration and core attributes** |
| --- |

**Tutorial I: Using DrumPID database**

**Tutorial part A**


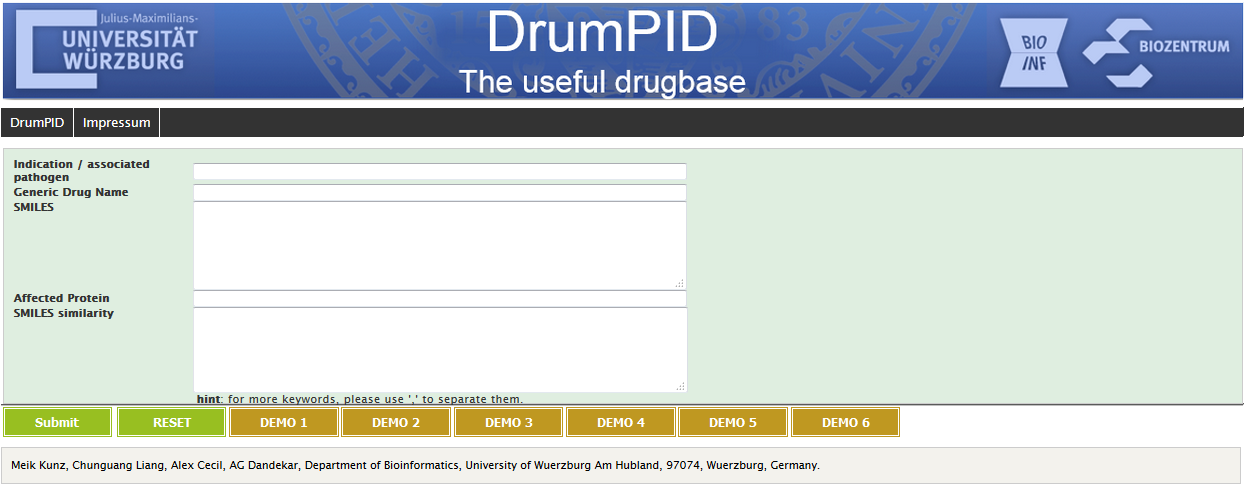


Tutorial A illustrates the Web interface for the DrumPID database. There are five search categories available: “Indication/associated pathogen”, “Generic Drug name”, “SMILES”, “Affected Protein” and “SMILES similarity”.

A query in the category “Indication/associated pathogen” will check against all indications of all drugs in the database. Thus it will be helpful to find out the best possible drug against a given pathological condition. It will also check against the organisms causing those conditions and gives all drugs against the pathogens to the user.

Queries in the category “Generic Drug name” are suitable if the generic brand names of the drugs are known to the users.

If neither the indication, nor the name nor the protein targets of a drug are known – or if the users want to check if his compound is chemically related to known drugs - the SMILES search category will be the best way to find similar drugs in the database. In order to use this function of the database, the canonical SMILES of the users' compound is mandatory.

For users searching for drugs which affect a specific protein and/or showing same functionality, the query should be posted in the category “Affected Protein” or “SMILES similarity”. For the “Affected Protein” search all drugs deposited in the database will be checked for their respective effects on target proteins, whereas the “SMILES similarity” will find similar substructure SMILES in all drugs based on the Tanimoto similarity score (>0.66).

*Of course a natural limitation of DrumPID is the amount and type of data stored, in particular searches will only work according to the key words given and suitable matches to data stored.*

In particular, check if the auto-completion works for the term you want to use, then the search will also give a good result from DrumPID. For a good search a detailed analysis combining several steps and key words is best. A further option besides querying with key words (indications, proteins etc.) are searches with SMILES strings according to chemical similarity. Thus use several terms and compare the results retrieved if you really want to get a good overview and choice of possible drugs, protein interactions and protein targets for your problem.

Multiple queries combining different categories are also accepted and multiple keywords in one category should be separated with comma (‘,’). Many user-friendly features have been implemented (e.g. Demo versions for each search category including a detailed description by moving the mouse above). Furthermore, DrumPID allows also concatenation of results and tools, this is illustrated in Tutorial III.

**Tutorial part B to E**


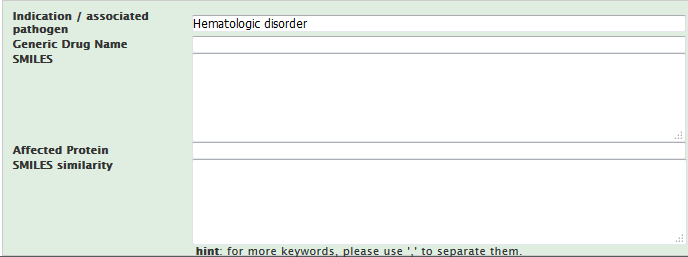


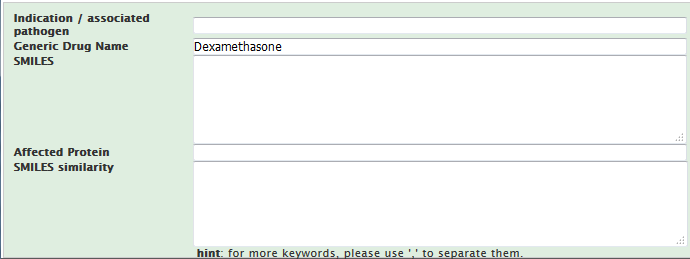


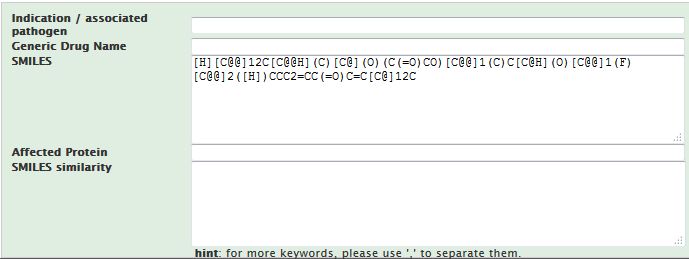


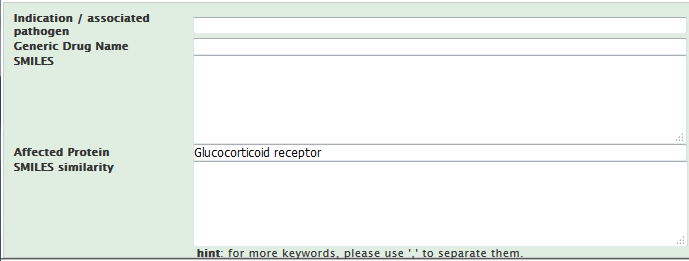


Tutorial parts B-E demonstrate various application examples. After giving the query description in the corresponding category and clicking the submit button below, the results of the search are immediately shown in the same page with the result entry number. When more than 50 matches are found, a summary page will be firstly displayed listing entry numbers as indexes (50 results in one page, and rest in another page). For these search examples, users get two results for Hematologic disorder, one for Dexamethasone, one for the SMILES search and 37 for the Glucocorticoid receptor search. Now, users can easily browse the results or, if demanded, choose to refine their search.

**Tutorial part F**


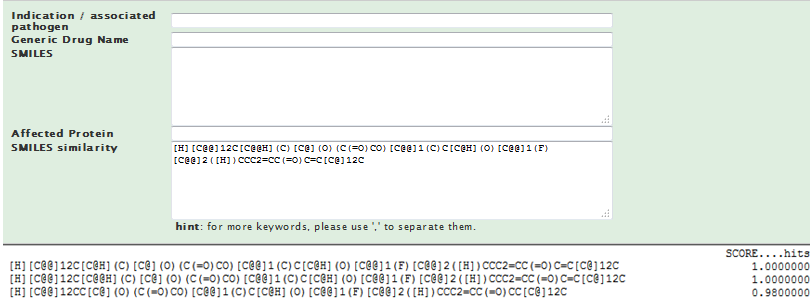


DrumPID includes a SMILES similarity search option based on the Tanimoto similarity scoring matrices (threshold >0.66). The example

[H][C@@]12C[C@@H](C)[C@](O)(C(=O)CO)[C@@]1(C)C[C@H](O)[C@@]1(F)[C@@]2([H])CCC2=CC(=O)C=C[C@]12C identifies 61 substrings similar to the input SMILES indicating that these drugs consist of a similar substructure SMILES (here top 3 hits are shown). Based on this, users can analyze the corresponding drugs e.g. to identify same targets as well as potential side-effects (the top 3 SMILES results are Dexamethasone, Betamethasone and Fludrocortisone (also called 9α-fluorocortisol or 9α-fluorohydrocortisone) which target the Glucocorticoid receptor; here not shown). Thus, this function is useful to obtain information of similar substructures in drugs allowing analysis of the drug functionality and development of new drug.

**Tutorial part G**


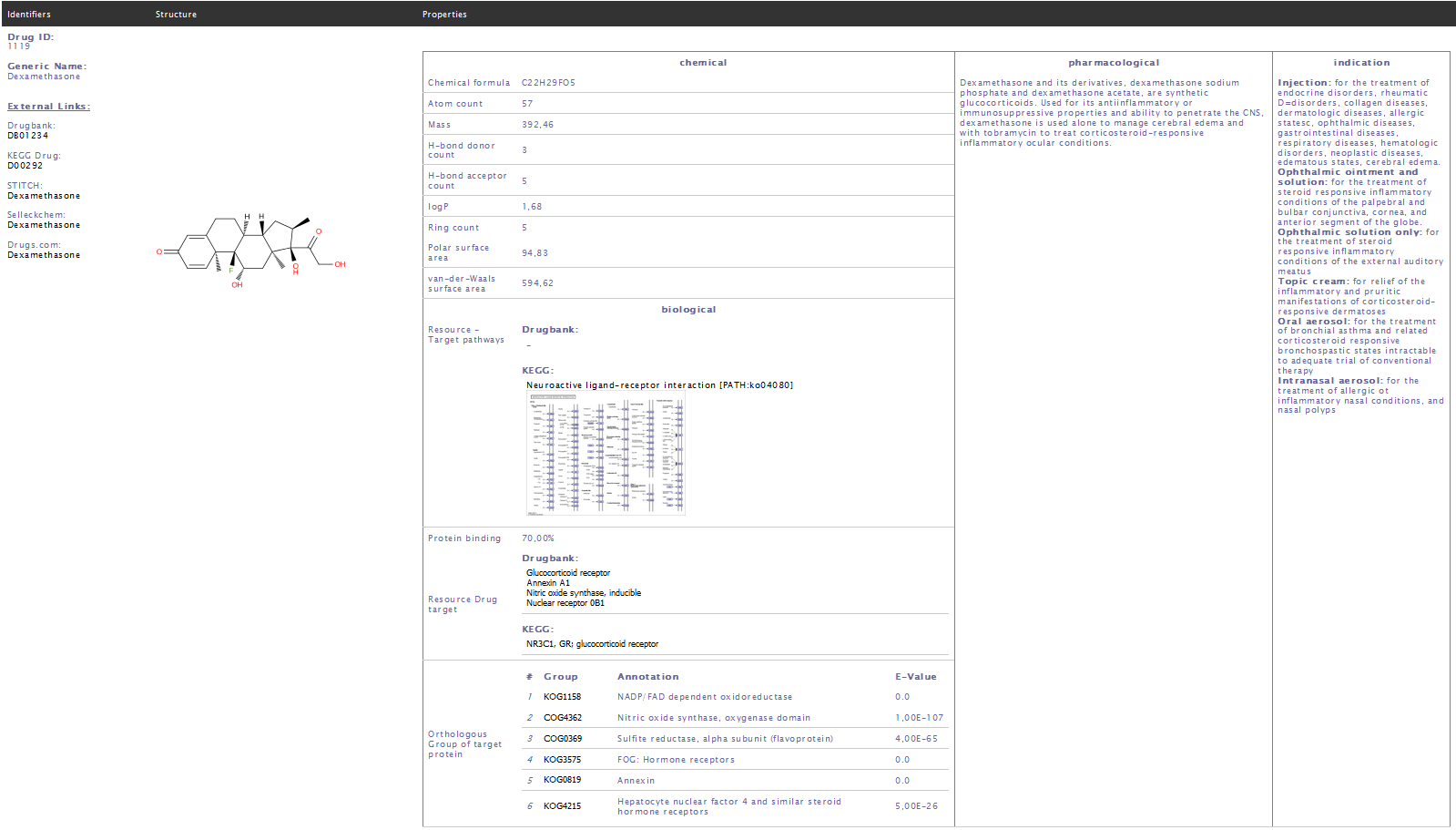


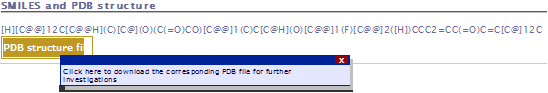


Tutorial part G represents the drug Dexamethasone which is one entry the user receives for each search example in Tutorials B-F. The result page is clearly structured and gives different outputs about general identifiers and information as well as chemical, biological, pharmacological and indication properties. The different chemical properties, e.g. the Lipinski's rule of five as important parameters for drug absorption, were calculated for all compounds in the database. Moreover, we also implemented a SMILES to PDB conversion function and users are able to download the corresponding PDB file or follow different crosslinks (see Tutorial II for more details).

**Tutorial part H**


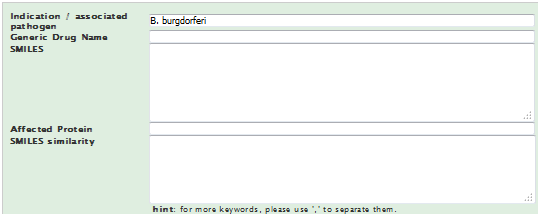


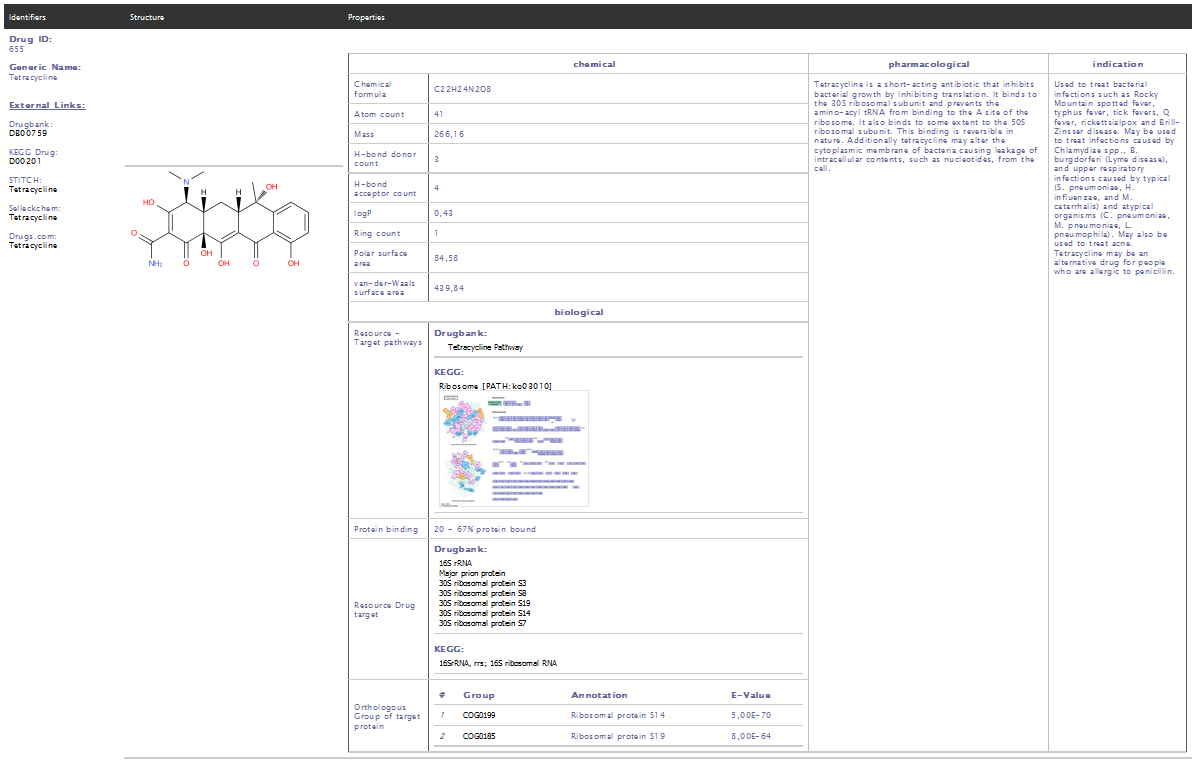


As mentioned in Tutorial A there is a search category in DrumPID for “Indications/associated pathogen”. Tutorial H demonstrates the search for a disease caused by the organism *B. burgdorferi* and yields tetracycline as treatment for this pathogen. Moreover, other possible indications for this drug as well as information about its mechanism of action (see column indication and pharmacological) are available here. Known drug target pathways are also presented to the users (here e.g. Ribosome pathway ko03010) and can again be investigated directly by hovering with the mouse over the pathway map and/or by following the links provided by DrumPID.

**Tutorial part J: Query concatenation**


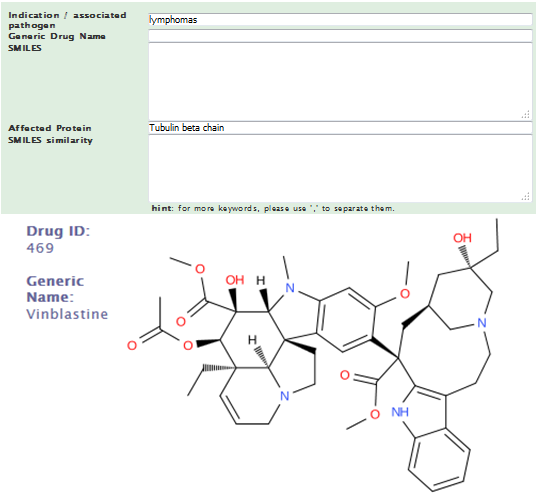


Tutorial J illustrates how queries joining more search categories at the same time (Example: “lymphomas” in the category “Indication/associated pathogen” and “Tubulin beta chain” in the category “Affected Protein”). In this example, DrumPID yields only one result (the drug: Vinblastine). However, DrumPID generated seven entries for Lymphomas and six when searching Tubulin beta chain (both not shown). In this case, query with more searching categories successfully reduces the output number and locates a better appropriate drug.

**Tutorial II: Putting the drug into its Protein Interaction context**

In the DrumPID database the drug pathways, targets and their protein interactions place the drug into its interaction context. We offer the following options:

1. The warehoused data on DrugBank and KEGG give for each drug the known protein target and the pathway including a corresponding source scheme and link. For the protein target, the corresponding cluster of orthologous groups is identified. The COG/KOGs are calculated comparing over all complete genomes all proteins belonging to the same gene family and label them with the same number to indicate that they belong to the same cluster of COG/KOG sequences. The COG/KOG helps to identify all proteins of this family occurring in the same organism. For a given drug, this indicates how easy the drug will reach besides the target protein other proteins belonging to the same family, which gives a good first estimate for potential side-effects (e.g. a large receptor family, where each receptor can also respond to the same drug). Furthermore, comparing the same COG/KOG over a range of organism allows to predict drug effects for a whole clade or even larger groups. This is for instance useful when estimating how broad an antibiotic drug will act. Furthermore, the COG/KOG allows also to see the complete variation of the target protein family over all organisms. Together with some pharmacological data (of course only if available) this allows a first estimate of quantitative structure-activity relationship for the target protein COG/KOG family (e.g. comparing N- and C-terminal variation in different organisms).
2. Further options include experimental and predicted protein-protein-interactions from well-known and not directly warehoused and crosslinked databases iHOP, HPRD, STRING and IMEx. The STRING database uses not only experimental validated protein-protein interactions but also a number of predictors to predict protein-protein interactions including gene context, correlation and neighborhood (all strong for prokaryotes) as well as correlations in expression profiling, text mining and large-scale interaction screens (strong in eukaryotes and man). Homology relationships make each of the predictions transferrable to homologous sequences. The inbuilt STRING database connection of DrumPID allows to rapidly set-up a network of all possible interactions as a first estimate of side-effects. Furthermore, if the user suspects an off-target interaction STRING offers by the multiple names option to put in a validated target and then estimates how many interactions in addition it takes to reach the suspected off-target protein. On the other hand, the IHOP database use interaction from the literature, however also allow the network generation based on experimental direct protein-protein-interaction data. The data on HPRD do not use predictions as STRING or clusters as COG/KOG but rather have hand curated, well surveyed data on direct, experimentally measured interactions occurring in human cells. Similarly, data from the IMEx consortium (International Molecular Exchange) contains non-redundant protein-protein interactions from several organism, which had expert curation from direct submissions or peer-reviewed journals such as IntAct, MINT and BioGRID. These are thus quite useful to estimate well known interactions in human cells and further organism, for instance to estimate early side-effects of a given drug.
3. The access to PlateletWeb carries this idea further and examines now tissue-specific protein interactions. Information for platelet protein interactions is given and compared to various interaction sources applying to other human cell types. Thus, users get information of platelet and all human interacting proteins allowing to explore the protein-protein-interactions in a tissue-specific context. Furthermore, known drug effects on any of the proteins shown to interact in this database are given as well as phosphorylation information for platelets as well as other human cell types. Tissue-specific information on protein interactions is also available from the STRING database and the GoSynthetic database by examining closer in the database the available information about the type of interaction and the protein function.
4. The GoSynthetic database uses again different prediction algorithm and clusters proteins (starting from the target protein) and their predicted interactions according to gene ontology terms and functional hierarchies. One option puts the drug and any found target protein of interest in the functional context of the protein functions known from a number of model organisms including man, *E.coli*, mouse. A second option studies the functional interactions found from an engineering perspective, giving suggestions for protein engineering experiments, functional links and system manipulation.
5. The link to the structure prediction tool AnDom. AnDom uses PSSMs to rapidly detect known structures for a given protein sequence. So any target protein of interest coming up in the analysis of the drug can be examined, how far structure information is available. As the output are direct links to PDB files, further detailed modeling of drug-protein interactions is facilitated by using this DrumPID link.

For each drug target we thus display the protein interactions according to the perspective of a number of databases and prediction software, giving a detailed view on involved protein interactions around a drug and its principal target and how to judge and compare these.


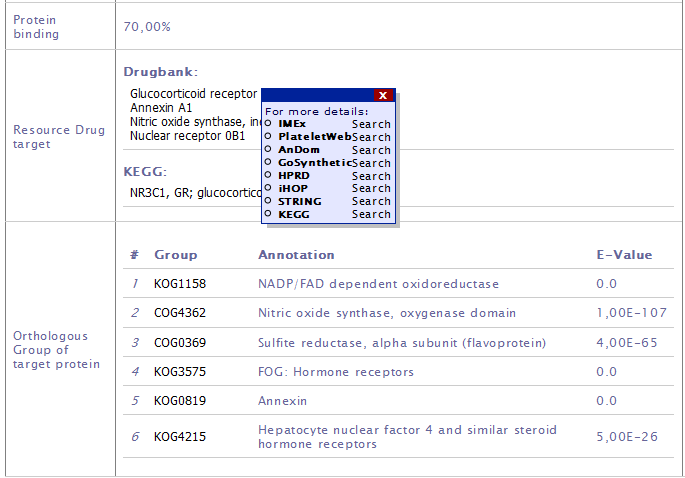


**Tutorial part K: Analyzing drug effects using COG/KOG information**

DrumPID warehoused the orthologous group in MySQL. In the example given, the group identified is the KOG3575 gene and protein family (one of the hormone receptors, found with high significant E-Value 0.0). The table available from DrumPID shows for the KOG3575 receptor family its distribution in different organism. The drug can act on all these receptors (e.g. Estrogen related receptor in mouse) and the user gets an overview on these multiple effects mediated by the COG/KOG family if the drug is given.


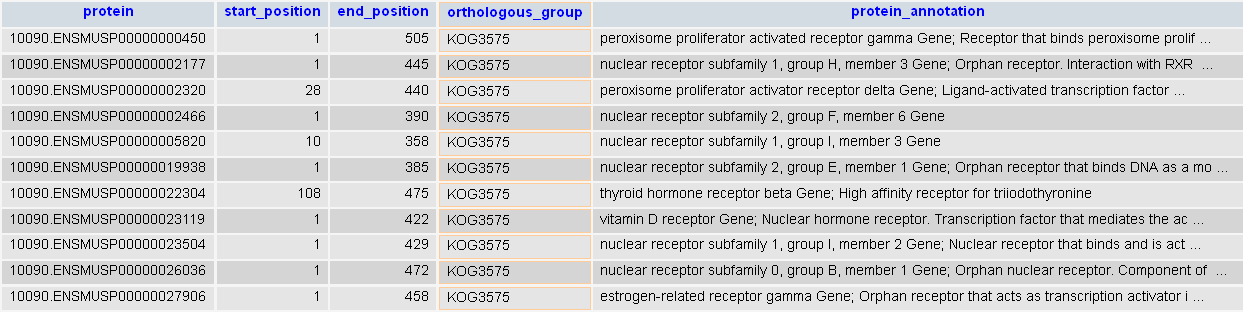


For analyzing drug effects with COG/KOG, users can also follow the links in DrumPID to STRING (only for COGs), EggNOG or GoSynthetic. There, further information about the ortholog gene are included, e.g. interactions.


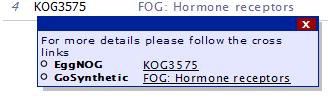


Regarding to the ortholog groups analysis of DrumPID, users get all relevant COG/KOGs and organisms for a drug and the option to find out the corresponding proteins as well as prediction of effects in different organisms, e.g. if antibiotic, which organisms have the target protein family (potential antibiotic action of the drugs) and which other not (prediction: no main target effects). Another alternative is also the estimate of binding opportunities and Quantitative structure-activity relationship (QSAR), in particular, if the analysis is coupled to structure files available via the AnDom link (see below).

**Tutorial L) Analyzing protein interactions with STRING**


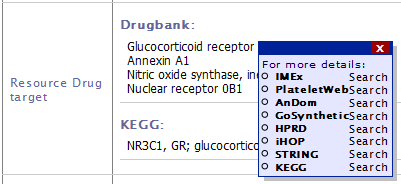


The STRING-database includes experimental validated protein-protein-interactions. In addition, STRING specializes on predicting protein interactions combining a number of large-scale experimental data as well as different interaction prediction tools (homology, text mining, co-expression, co-occurrence and neighborhood). Therefore, users get for a drug target further interactions by moving the mouse above to the corresponding DrumPID output (e.g. Glucocorticoid receptor). Furthermore, users can select an organisms or search against all organism (auto-detect).


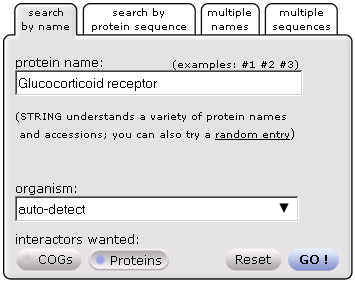


After clicking to the Go button, query results are shown (here only two) and users can follow the entry of interest (here for NR3C1 in human).


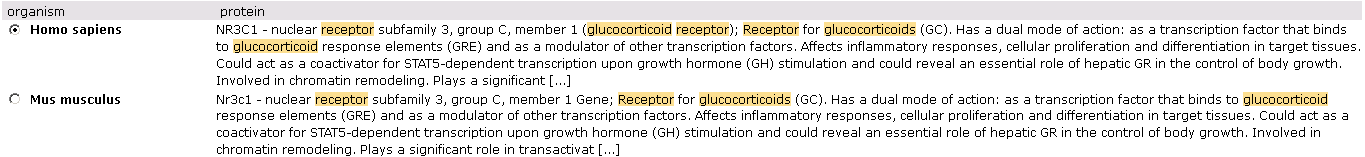


After this, a protein interaction network is given as well as further information about the proteins and the interaction type (e.g. violet edges are experimental and light green text mining; here only interaction for NR3C1 is shown).

**
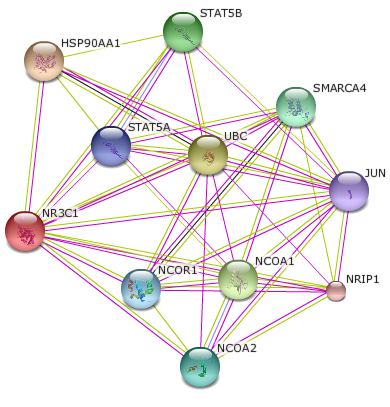
**

**Tutorial M) Analyzing protein interactions and drug effects with HPRD**

To find out which interactions occur in the patient users can follow the crosslink to HPRD, iHOP and IMEx (here shown for HPRD).


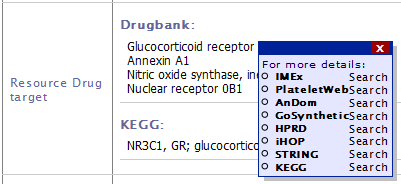


The HPRD database assembles hand curated data on verified human protein-protein interactions (see above). By moving the mouse above to the corresponding output (e.g. Glucocorticoid receptor) users can follow the crosslink to HPRD by clicking Search and the query results are shown in a new window. For the Glucocorticoid receptor users get two entries including an overview.


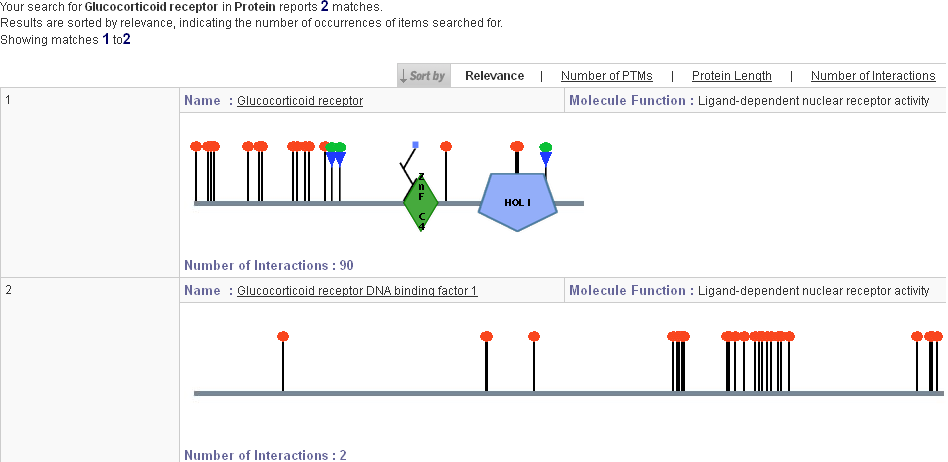


By clicking to Name (Glucocorticoid receptor, first entry) a detailed list of the protein interactions as well as further information (e.g. experimental type) of the corresponding entry is given. Therefore, DrumPID allows analyzing and prediction of drug effects as well as side-effects.


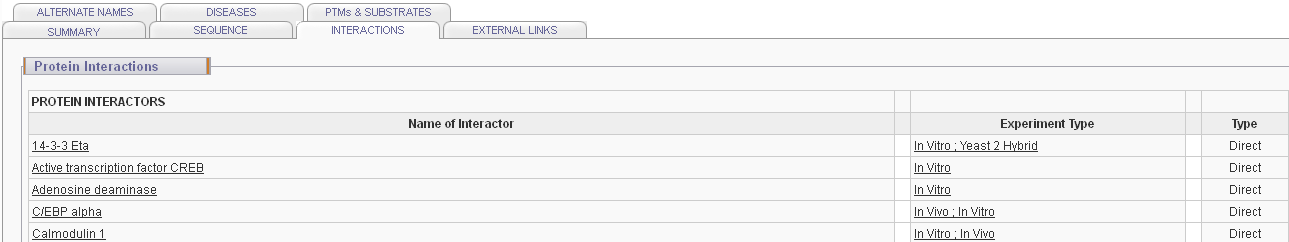


**Tutorial part N: Analyzing protein interactions and drug effects with the PlateletWeb**


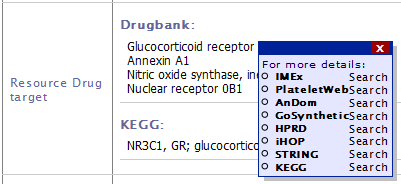


Tissue-specific protein interactions as well as known drug effects on any protein in the interaction network are available from the PlateletWeb crosslink. The database thus includes tissue-specific expression datasets and many cardiovascular drugs including knowledge on cardiovascular side-effects. Tissue specific protein interactions allow minimizing side-effects, in particular platelet versus other tissues. Moreover, interaction predictions for all human tissues are implemented as well as concrete Phosphorylation-information and all known drug interactions for all human proteins are incorporated. Clicking in DrumPID to the PlateletWeb Search, query results are shown in a new window (here five for the Glucocorticoid receptor). For each entry an overview is given (e.g. about the detection level and the number of interactions) and users can follow the interactions of interest by clicking to the number of total interacting proteins in the Description column (7 for the entry glucocorticoid receptor DNA binding factor 1).


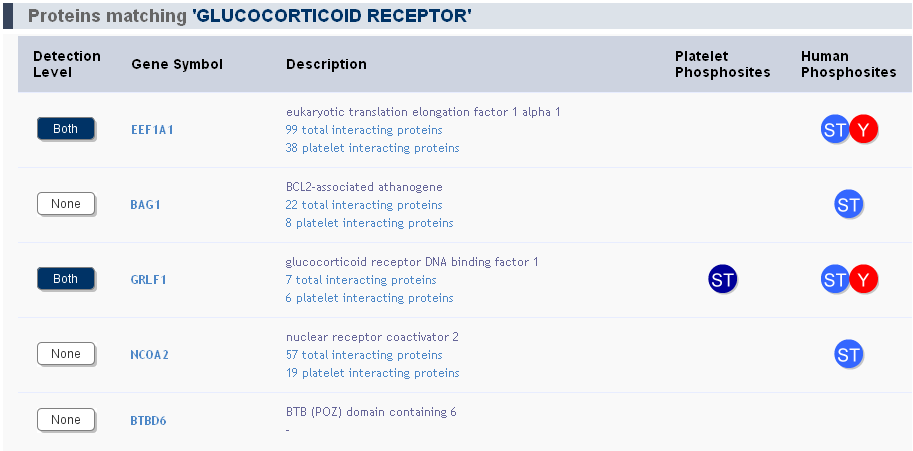


Now, users get all interaction partners including their interactions and can also view the interactions in different network formats (network download, e.g. as pdf or cytoscape session, is available).


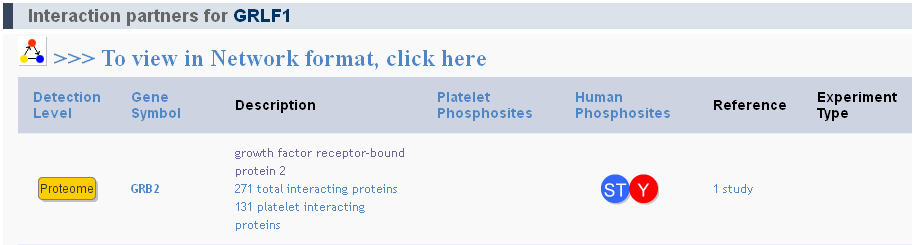


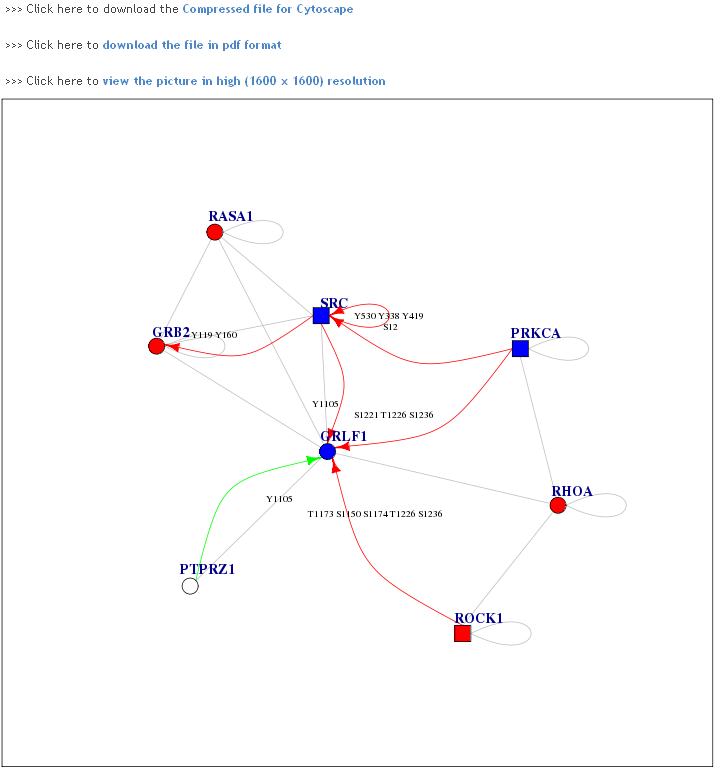


**Tutorial part O: Analyzing drug effects with GoSynthetic**

With DrumPID users can follow also the crosslink to our in-house GoSynthetic database.


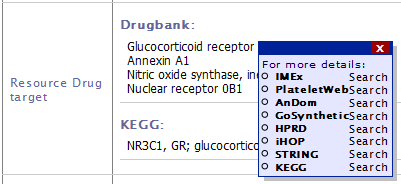


There, analyzing of drug effects including the hierarchical functional and gene onthology categories help to better analyze a drug target and its functional interactions according to gene onthology and gene functions. With GoSynthetic users can search systematically for protein targets with a specific function in mind and analyze a protein interaction network around a drug regarding involved functions. Moreover, these categories help also to reveal side-effects and make a prediction of affected subnetworks and of drug combination effects. A bridging function is implemented in DrumPID such that also the corresponding target sequence of the target sequence of interest is analyzed by GoSynthetic. A model organism (in this example Homo sapiens) of choice can be specified, next functional hierarchy, interaction networks and clustering of involved functions is examined.


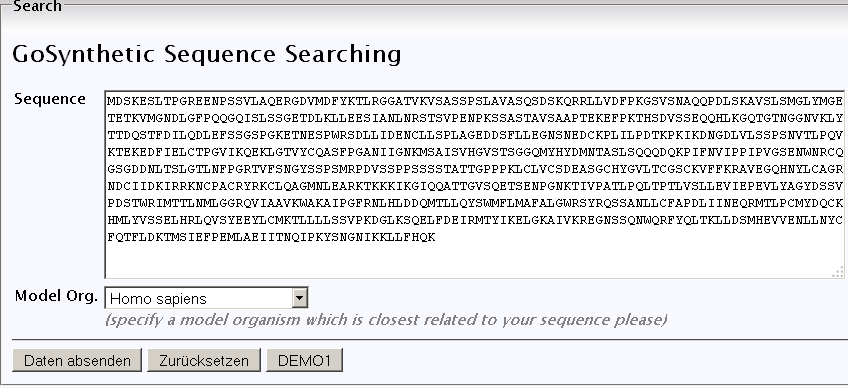


Results including different parameters, e.g. GO-ID, Description and a pie chart view, are given, lists of descriptions list all functions involved in the sub-network analyzed (here for glucocorticoid receptor activity).


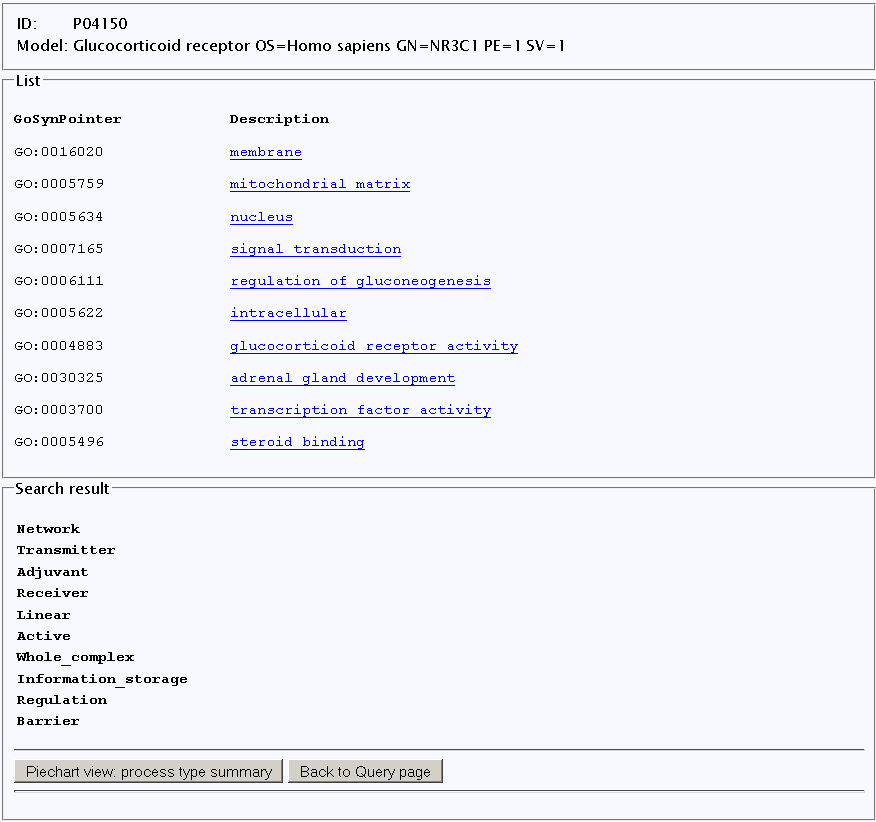


In the example given, users obtain the functional relation (biological function as controller and technical function as Receiver) including interactions and can also download the interaction network file for cytoscape.


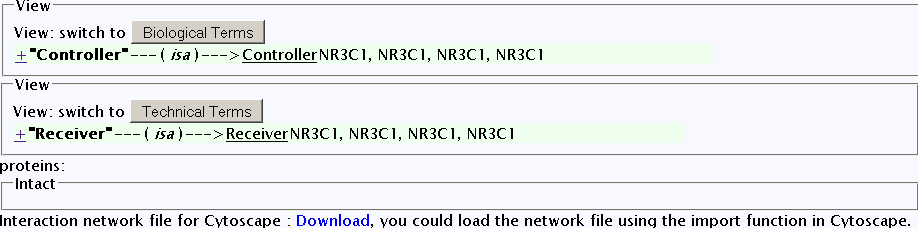


Another option is the usage of the VRelations view with GoSynthetic, showing all interactions graphically including their functional clustering. This allows to scan for side-effects and to optimize the function profile of the drug.

**Tutorial part P: Analyzing drug effects with AnDom**

For a given target in DrumPID, users can identify all domains including structure information as well as the PDB structure file (available for download) for docking and drug optimization. Furthermore, the finding of a potential structure for a drug is also feasible in this context. This works for every protein target sequence which has at least little fact with 3D homology.


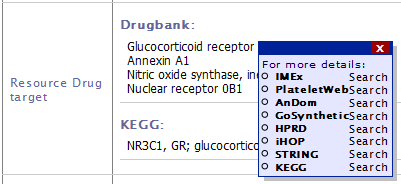


The crosslink to our in-house AnDom server opens a new window and the implemented bridging function puts the sequence (in this example for Glucocorticoid receptor) into the search field.


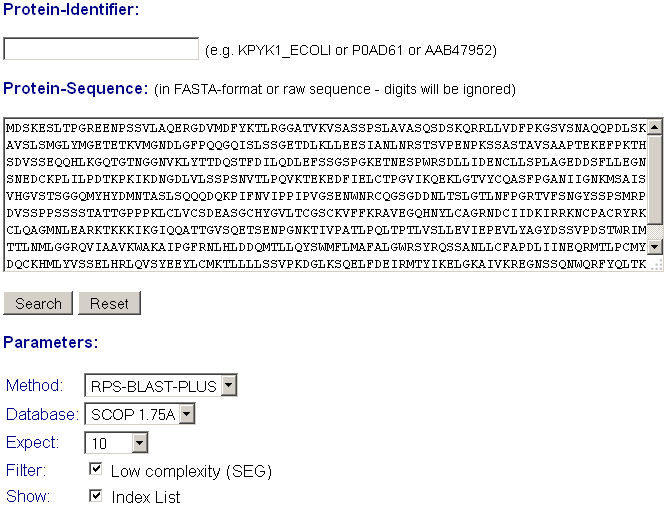


Users can select between different parameters (e.g. Method and Database) and click to the Search button for analyzing the sequence. The Search result will be immediately displayed including structural classification information, e.g. SCOP identification, description and E-Value (please note, here only four results shown).


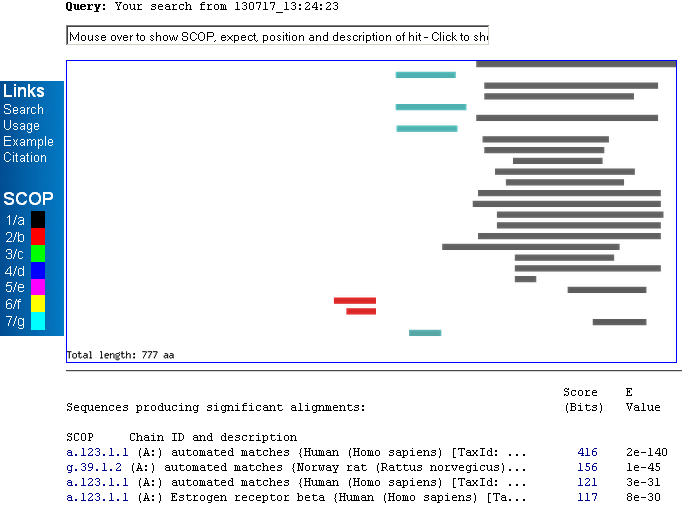


Users get the alignment by scroll down or clicking to the Score value (here for Estrogen receptor beta is presented).


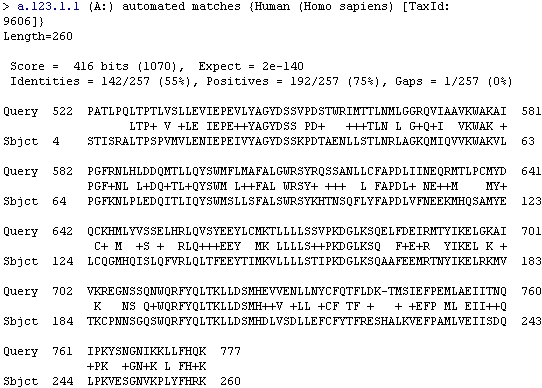


By clicking to the corresponding SCOP-ID, structural information also available and makes further domain investigations easily possible (here about the Nuclear receptor ligand-binding domain not shown).

**Tutorial III: Application study anti-fungal drugs**

Aspergillus fumigatus causes Aspergillosis, a life-threatening infection in immune compromised patients (e.g. transplantation patients). Treatment is challenging as the fungus is an eukaryotic organism similar to the human patients. Therapeutic agents often show thus toxic effects in humans. We study Aspergillus fumigatus metabolism in an effort to identify new drug targets. Also for this task DrumPID can give helpful hints as part of a comprehensive effort. Screening DrumPID identifies different standard antifungal drugs. For a comprehensive overview, data entries from different key words are collected, for instance “Aspergillus” (under indication category) identifies the seven drugs, e.g. Anidulafungin and Posaconazole, both used for Aspergillus treatment through acting on common pathways by inhibition of fungal cell wall synthesis, blocking steroid biosynthesis. This is a narrow search, focussing only on well-established Aspergillus drugs contained in the database. For comparison, the key word „antifungals“ retrieves five drugs and the general keyword „fungal“ retrieves 32 drugs including those targeting Cytochrome P450 (a second hit on the steroid biosynthesis pathway).

DrumPID yields also drug information querying concrete pathways. Thus for the already explored antifungal target pathway ergosterol, DrumPID identifies six drugs which all are used in the clinic for antifungal treatment, e.g. Amphotericin B and Natamycin. However, for many other metabolic pathways in most cases a drug (in our example an antifungal drug) is not known. In such cases the implemented COG/KOGs search in DrumPID enables users to find orthologs among species to develop new potential antibiotics for further experimental test. We illustrate this for a more challenging target protein from our analysis of fungal metabolism, riboflavin synthase.

Potential antibiotics against *Aspergillus fumigatus* infection using COG/KOGs information

For riboflavin kinase DrumPID correctly reports the substrate riboflavin as interacting compound or drug (under indication category), also known as vitamin B2, and sold as nutraceutical. However, we stated already that for many fungal pathways the closeness to human proteins (orthology) is a challenge. DrumPID is useful to develop also in this case new therapeutic agents, which target only the fungal riboflavin kinase with negligible effects on the human kinase. For this, based on our implemented orthologous group analysis users get all relevant COG/KOGs for the drug targets, here this is the KOG3110 (riboflavin kinase).


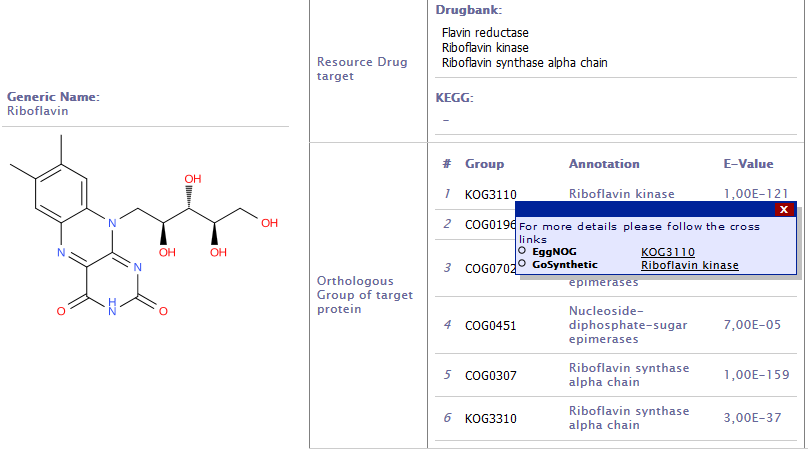


According to EggNOG database (following link in DrumPID) users can obtain the riboflavin kinase protein in human (RFK) and different *Aspergillus* strains (e.g. *Aspergillus terreus* ATEG_06773, *Aspergillus niger* FMN1) (here not shown). Based on the protein sequences, users can find the corresponding riboflavin kinase in *Aspergillus fumigatus* (Af293) using BLAST algorithm (here not shown), in which a protein sequence alignment using BLAST shows only sequence identity around 38-41% between the three *Aspergillus* strains and human (top; query sequence=human), whereas in-between similarity of both *Aspergillus* strains to *Aspergillus* *fumigatus* is over 70% (bottom; query sequence=*Aspergillus* *fumigatus*), indicating flexible regions between human and *Aspergillus* for drug-target modeling.


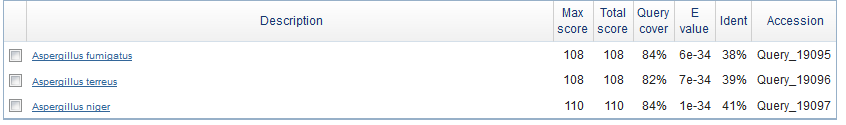

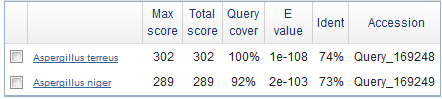


However, for drug development functional binding sites are important. According to UniProt database (here not shown), riboflavin kinase of human and the three *Aspergillus* strains contain similar active site (see star below E) and metal binding site (see star above T). Interestingly, integrating this information into the sequence alignment from BLAST indicates that the flanking regions around the sites are dissimilar (flanking region around active site in violet; flanking region around metal binding site in blue; Query=human, Subject= *Aspergillus*). Moreover, there exist also two gap sequence regions (labeled in red and green) between *Aspergillus* and human. Thus, this indicates not only different regions around the functional sites but also additional flexible regions between human and *Aspergillus* for drug-target modeling to specifically target only the fungus-specific protein but not the human kinase protein.


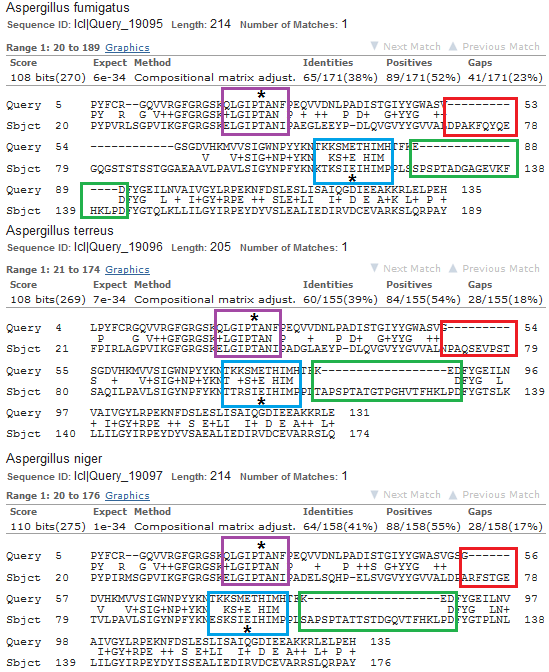


Moreover, an internal sequence comparison between the three *Aspergillus* strains (in-between sequence identity >70%) shows that the green gap sequence region from the comparison to the human version occurs in all three fungi (labeled in green; useful to be targeted by a broad antifungal drug), whereas the red gap sequence region differs between the *Aspergillus* strains (Query= *Aspergillus fumigatus*, Subject=two other *Aspergillus* strains).


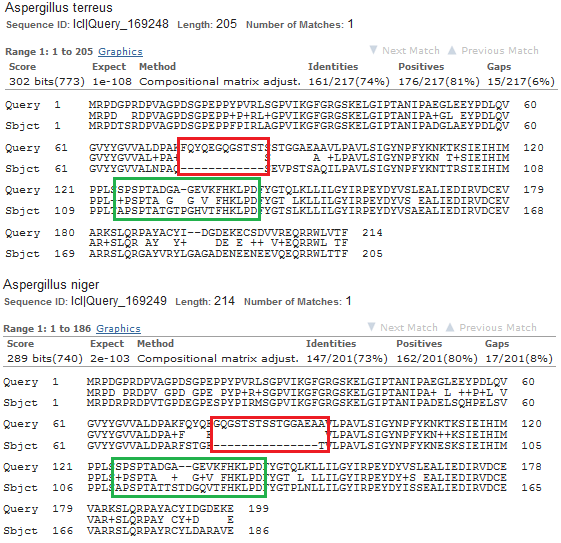


Thus, using all these information as well as the 3D structure of Riboflavin (directly from DrumPID) and a homology model of *Aspergillus fumigatus* riboflavin kinase (three structures available in PDB: human, Schizosaccharomyces pombe and archaebacterial version), there is a solid basis to study *in silico* the drug-protein interaction of modified riboflavins, e.g. replacement or additional side-groups, so that the modified riboflavin derivative specifically binds the *Aspergillus fumigatus* riboflavin kinase without toxic effects in the human patient.

**Declarations and database core attributes**

• DrumPID is freely available without any registration limits.

• DrumPID will be maintained under the URL [http://drumpid.bioapps.biozentrum.uni-wuerzburg.de](http://drumpid.bioapps.biozentrum.uni-wuerzburg.de/) and constantly updated each month.

• DrumPID consists of 1383 drugs including 4078 Orthologous groups (linked to 993 unique COG/KOG with 21120 orthologous genes in 67 different organism) and 4951 proteins (linked to targets and protein interactions).

• All data are available and downloads and Hyperlinks will be integrated in the URL, after acceptance we will also make the existing >5000 approved and non-approved FDA drugs available.

• A supplementary file is included listing the **database core attributes according to the BioDBcore** standards (<http://biocurator.org/biodbcore.shtml>).

1. Database name: DrumPID database
2. Main resource: http://drumpid.bioapps.biozentrum.uni-wuerzburg.de
3. Contact information (e-mail; postal mail):

Prof. Thomas Dandekar: [dandekar@biozentrum.uni-wuerzburg.de](mailto:dandekar@biozentrum.uni-wuerzburg.de)

Postal Address: Bioinformatics, Biocenter,
University of Wuerzburg Am Hubland,
D-97074, Bayern, Germany

1. Date resource established (2011)
2. Conditions of use: Free and publicly open.
3. Scope: data types captured, curation policy, standards used

- targets

- pathways

- protein interactions

- Orthologous groups

1. Standards: MIs, Data formats, terminologies

- COG/KOG terms

- Taxonomic coverage: *Homo sapiens*

- All listed prokaryotes and eukaryotes according to the Orthologous groups

1. Data accessibility/output options

- Plain-text

- HTML

- Structure image file

- SMILES/PDB structure file

1. Data release frequency: Monthly updated
2. Versioning period and access to historical files (not provided)
3. Documentation available
4. Supplemental files and tutorial available
5. User support options
6. Authorized user support
7. Data submission policy (not provided)
8. Resource's Wikipedia URL (not provided)
9. Tools available (not provided)

**References and links:**

🡪 PlateletWeb: <http://plateletweb.bioapps.biozentrum.uni-wuerzburg.de/plateletweb.php>

- Boyanova, D. et al. (2012) PlateletWeb: a systems biological analysis of signaling networks in human platelets. *Blood*, **119**(3), e22-34.

🡪 KEGG: http://www.genome.jp/kegg/

- Kanehisa., M. *et al.* (2012) KEGG for integration and interpretation of large-scale molecular datasets. *Nucleic Acids Res.* **40**, D109-D114.

🡪 STRING: <http://string-db.org/>

- Franceschini, A. *et al.* (2013) Szklarczyk, D. et al. (2015) STRING v10: protein-protein interaction networks, integrated over the tree of life. Nucleic Acids Res., 43 (Database issue):D447-52. doi: 10.1093/nar/gku1003.

🡪 IHOP: <http://www.ihop-net.org/UniPub/iHOP/>

- Hoffmann, R. and Valencia, A. (2004) A Gene Network for Navigating the Literature. *Nature Genetics* **36**, 664.

🡪 AnDOM: <http://andom.bioapps.biozentrum.uni-wuerzburg.de/index_new.html>

- Schmidt, S., *et al.* (2002) A versatile structural domain analysis server using profile weight matrices. *J Chem Inf Comput Sci.* **42** (2), 405-7.

🡪 COG/KOG: <http://www.ncbi.nlm.nih.gov/COG/>

- Tatusov, R.L., *et al.* (2003) The COG database: an updated version includes eukaryotes. *BMC Bioinformatics.* **4**, 41.

🡪 eggNOG: http://eggnogdb.embl.de/#/app/home

- Huerta-Cepas, J. *et al.* (2016) eggNOG 4.5: a hierarchical orthology framework with improved functional annotations for eukaryotic, prokaryotic and viral sequences. *Nucl. Acids Res.*,**44** (D1): D286-D293. doi: 10.1093/nar/gkv1248.

🡪 HPRD: <http://www.hprd.org/>

- Prasad, K.T.S., *et al.* (2009) Human Protein Reference Database - 2009 Update. *Nucleic Acids Res*. **37**, D767-72.

🡪 IMEx: http://www.imexconsortium.org/

- Orchard, S., *et al.* (2012) Protein interaction data curation: the International Molecular Exchange (IMEx) consortium. *Nat Methods* **9**, 345-350.

🡪 GoSynthetic: <http://gosyn.bioapps.biozentrum.uni-wuerzburg.de/index.php>

- Liang, C., et al. (2013) GoSynthetic database tool to analyse natural and engineered molecular processes. Database (Oxford), 2013:bat043. doi: 10.1093/database/bat043.
